# Phenotype and fate of liver-resident CD8 T cells during acute and chronic hepacivirus infection

**DOI:** 10.1371/journal.ppat.1011697

**Published:** 2023-10-09

**Authors:** Piyush Dravid, Satyapramod Murthy, Zayed Attia, Cole Cassady, Rahul Chandra, Sheetal Trivedi, Ashish Vyas, John Gridley, Brantley Holland, Anuradha Kumari, Arash Grakoui, John M. Cullen, Christopher M. Walker, Himanshu Sharma, Amit Kapoor

**Affiliations:** 1 Center for Vaccines and Immunity, Abigail Wexner Research Institute at Nationwide Children’s Hospital, Columbus, Ohio, United States of America; 2 Emory National Primate Research Center, Division of Microbiology and Immunology, Emory Vaccine Center, Emory University School of Medicine, Division of Infectious Diseases, Atlanta, Georgia, United States of America; 3 North Carolina State University College of Veterinary Medicine, Raleigh, North Carolina, United States of America; 4 Department of Pediatrics, College of Medicine and Public Health, The Ohio State University, Columbus, Ohio, United States of America; University of Alabama at Birmingham, UNITED STATES

## Abstract

Immune correlates of hepatitis C virus (HCV) clearance and control remain poorly defined due to the lack of an informative animal model. We recently described acute and chronic rodent HCV-like virus (RHV) infections in lab mice. Here, we developed MHC class I and class II tetramers to characterize the serial changes in RHV-specific CD8 and CD4 T cells during acute and chronic infection in C57BL/6J mice. RHV infection induced rapid expansion of T cells targeting viral structural and nonstructural proteins. After virus clearance, the virus-specific T cells transitioned from effectors to long-lived liver-resident memory T cells (T_RM_). The effector and memory CD8 and CD4 T cells primarily produced Th1 cytokines, IFN-γ, TNF-α, and IL-2, upon *ex vivo* antigen stimulation, and their phenotype and transcriptome differed significantly between the liver and spleen. Rapid clearance of RHV reinfection coincided with the proliferation of virus-specific CD8 T_RM_ cells in the liver. Chronic RHV infection was associated with the exhaustion of CD8 T cells (Tex) and the development of severe liver diseases. Interestingly, the virus-specific CD8 Tex cells continued proliferation in the liver despite the persistent high-titer viremia and retained partial antiviral functions, as evident from their ability to degranulate and produce IFN-γ upon *ex vivo* antigen stimulation. Thus, RHV infection in mice provides a unique model to study the function and fate of liver-resident T cells during acute and chronic hepatotropic infection.

## Author summary

Approximately 60–80% of humans fail to clear the hepatitis C virus infection, and the mechanism of HCV persistence and immune evasion remains elusive. T cells play an essential role in the control and clearance of HCV infection in humans and chimpanzees. Recently, we developed lab mouse and rat models for rodent HCV-like virus (RHV). Further development of these new rodent models is highly significant due to the lack of relevant animal models to study HCV immunity and pathogenesis. Here, we developed and used mouse MHC class I and class II tetramers to characterize the RHV-specific effector and memory T cells during acute and chronic RHV infection in immunocompetent lab mice. The control and clearance of primary and secondary RHV infection were associated with the expansion of liver-resident T cells expressing cytotoxic markers and antiviral cytokines. Chronic infection resulted in the exhaustion of T cells and the development of severe liver diseases. We defined the fundamental characteristics and unique transcriptome signature of liver-resident RHV-specific CD8 T cells during acute and chronic hepacivirus infection. Thus, our data and the newly developed mouse MHC class I and class II tetramers will allow the RHV mouse model to identify the liver-specific responses that shape the nature of liver-resident T cells and the divergent outcomes of hepacivirus infection and pathogenesis.

## Introduction

Hepatitis C virus (HCV) chronically infects 71 million people worldwide, contributing to ~400,000 deaths annually [1]. Within the United States, HCV is a leading cause of mortality by an infectious pathogen exceeding that of HIV and tuberculosis combined. Transmission rates are rising due to a resurgence in injection drug use fueled by an ongoing opioid epidemic [2,3]. Despite the advent of all-oral, direct-acting antiviral (DAA) regimens that cure most infections, significant barriers to HCV eradication remain, including the high cost of therapy, inadequate infection surveillance programs, and poor treatment adherence within difficult-to-treat patient groups [4,5]. Additionally, individuals cured by DAA therapy remain susceptible to HCV reinfection [6,7], which could complicate eliminating the virus from high-risk populations, such as injection drug users. Thus, a preventive vaccine, effective in HCV-naïve and DAA-cured individuals, will ultimately be needed to help curb HCV transmission and achieve global elimination goals [8,9].

A comprehensive knowledge of immune correlates of protection against HCV infection is critical for designing an effective vaccine [10]. However, the host-specificity of HCV and the consequent lack of an animal model resulted in a poor understanding of antiviral immunity and immune correlates of protection [9–11]. In addition to common hepatitis viruses A to E, several other viruses can infect or induce liver immunopathologies. The unique microenvironment of the liver balances immunity and tolerance by generating a robust immune response while avoiding excessive inflammation and tissue damage [12]. Although the initial immune response to viral infection in the liver is often robust, supported by a rapid innate immune response and subsequent expansion of pathogen-specific T cells, early T cell exhaustion or depletion frequently leads to the establishment of chronic infection [12–15]. Limited access to the human liver, particularly during the early stages of infection, has restricted the identification of immune responses that determine the clearance or persistence of hepatotropic viruses [16,17]. Similar studies in nonhuman primates were limited by access to sufficient animals and ethical concerns [18,19]. Thus, developing an immunocompetent animal model to study acute and chronic viral infection of the liver is fundamental to defining the nature of immunity required to control or prevent chronic viral infection [20]. However, lab mice and rats are not susceptible to infection by human hepatitis viruses, and thus an alternative is to use rodent viruses that are homologs of human viruses and naturally recapitulate their infection and immunopathogenesis [11].

The isolation of HCV-like rodent hepacivirus (RHV) from wild rats has enabled the development of informative surrogate models for HCV [21–24]. RHV differs from HCV in sequence, but the shared genomic features and organization, polyprotein cleavage pattern, liver tropism, and DAA susceptibility make RHV a highly relevant HCV surrogate model [23,25,26]. RHV establishes exclusively hepatotropic infection in normal lab mice and rats with immunological characteristics resembling HCV infection in humans [22,23,27,28]. Furthermore, like HCV infection in humans and chimpanzees [16–18,29,30], T cell immunity plays an essential role in the clearance of RHV infection in rat and mouse models [22,27,31]. Chronic life-long RHV infection in immunocompetent rats is associated with subversion of T cell immunity [31]. In contrast, functional T cells protect against RHV infection and reinfection in mice [27]. Notably, vaccination using the recombinant adenoviral vectors encoding the RHV NS3-5B proteins reduced the incidence of persistent infection in rats after the homologous RHV challenge [22,24] and prolonged or persistent RHV infection after antibody-mediated depletion of CD8 or CD4 T cells in vaccinated rats and reduced efficacy of the vaccine against RHV encoding mutations in dominant MHC class I epitopes further established a critical role for T cells in vaccine-mediated protection [22,28].

However, despite the unique suitability of the RHV-rat model for HCV vaccine research [22,28], the scarcity of molecular, genetic, and immunological reagents for laboratory rats restricts a detailed analysis of virus-host interactions that determine the fate of primary and secondary virus infection [10]. Thus, the parallel development of a laboratory mouse model to study acute and chronic RHV infection would gain knowledge that remained obscure due to the lack of a relevant animal model for HCV [27]. Importantly, spontaneous clearance of RHV infection in the mouse model offers a unique opportunity to characterize the nature of immunity, specifically the formation and features of virus-specific memory T cells, that can effectively control hepacivirus infection and reinfection. Here, we analyzed the nature and breadth of T cell immunity induced by RHV infection in immunocompetent adult lab mice. We developed novel mouse MHC class I and class II tetramers to visualize and characterize RHV-specific CD8 and CD4 T cells in C57BL/6J mice. We defined the serial changes in RHV-specific liver-resident memory T (T_RM_) cells during virus clearance and persistent infection. Additionally, we determined the response of memory T cells leading to control and clearance of RHV reinfection and identified the unique transcriptome signature of RHV-specific liver CD8 T_RM_ cells.

## Results

### Nature of T cell expansion in the liver during RHV infection and clearance

**Fig 1:**
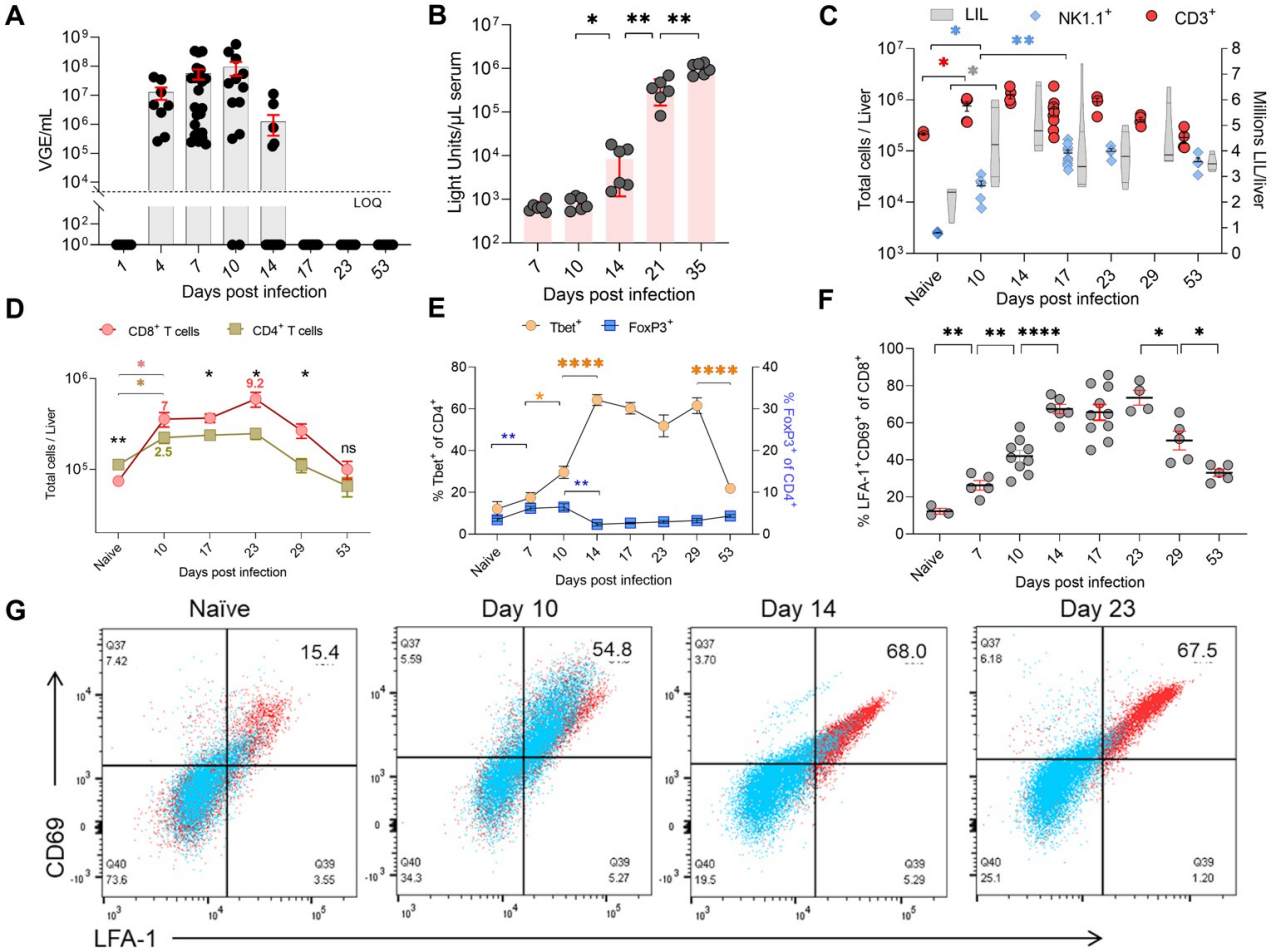
RHV clearance is associated with the expansion of T cells and the formation of CD8 T_RM_ cells in the liver. **(A)** The course of RHV viremia in 6–8 weeks old C57BL/6J mice. A few mice cleared the viremia earlier than most others, between days 10 to 17 p.i. The dotted line indicates the limit of quantification (LOQ) of RT-PCR assay. VGE is virus genomes equivalent per ml of serum. **(B)** Titers of anti-NS3 IgG antibodies were measured using LIPS assay in the serum samples of infected mice. **(C)** Serial analysis of total liver-infiltrating leukocytes (LIL), NK (NK1.1^+^), and T (CD3^+^) cells in the liver of infected mice. **(D)** Absolute numbers of CD4 and CD8 T cells per liver in the RHV infected mice. The numbers on the graph indicate the fold increase relative to the naive mice. Black asterisks indicate significant differences between CD4 and CD8 T cells at the same time point, and colored asterisks indicate significant differences between CD8 or CD4 T cells between different time points. **(E)** Serial analysis of CD4 T cell differentiation to compare the proportion of FoxP3^+^ Tregs and T-bet^+^ CD4 T cells in the liver of infected mice. **(F)** The expansion of CD8 T cells coexpressing tissue-retention markers CD69 and LFA-1 in the liver after RHV infection. **(G)** Representative flow plots of liver-infiltrating CD8 T cells expressing CD62L (blue) overlayed on total CD8 T cells (red) gated for the expression of CD69 and LFA-1 in infected mice on different days after RHV infection. The data are shown for individual mice or as mean ± SEM of 4–11 mice done in >2 independent experiments. All statistical analysis was done in GraphPad PRISM 9.0.0. using a two-tailed unpaired t-test, and only significant p-values are shown, where p-value <0.05 (*), <0.01 (**), <0.001 (***), and <0.0001 (****).

Intravenous injection of RHV (10^4^ virus genome equivalent, VGE) in 6–8 weeks old C57BL/6J mice produced robust viremia that was cleared within two weeks in most mice (Fig 1A). Notably, the titers and duration of viremia differed widely between individual mice even when these were inbred (Fig 1A). Although, unlike our earlier study that used a heterogenous RHV stock [27], here we used a genetically homogenous stock rescued by injecting transcripts of a genome clone of RHVrn-1 in rats (GenBank: KX905133.1) [23]. All infected mice seroconverted, as evident from the significant increase in the titers of anti-RHV NS3 antibodies between days 14 to 35 p.i. (Fig 1B). As reported earlier [27], flow cytometric analysis showed significant increases in the numbers of total leukocytes, NK, and T cells in the liver of mice after RHV infection (Fig 1C). Notably, we determined earlier that NK cells are dispensable, while T cells are indispensable for clearance of RHV infection in mice [27]. Thus, we focused here on characterizing T cells in the liver after RHV infection. Comparative analysis of CD8 and CD4 T cells in the liver showed that while both T cell subsets significantly increased in absolute numbers after RHV infection, the CD8 T cells increased or expanded more than the CD4 T cells (7–9 fold vs. 2.5 fold) (Fig 1D). Analysis of transcription factors in intrahepatic CD4 T cells indicated a simultaneous increase in proportions of T-bet^+^ and FOXP3^+^ CD4 T cells immediately after infection, followed by significant contraction of FOXP3^+^ CD4 T cells between day 10 and 14 p.i., while the expansion of T-bet^+^ CD4 T cells continued until day 14 (Fig 1E). Furthermore, more than half of the CD4 T cells in the liver were T-bet^+^ from day 14 to 29 p.i., and after that contracted rapidly. To determine the nature of CD8 T cells in the liver of infected mice, we compared their phenotype before, during, and after clearance of RHV infection. Immediately after RHV infection, the liver CD8 T cells co-expressing LFA-1 and CD69 expanded >2-4-fold [32] (Fig 1F). Interestingly, these CD8 T cells significantly expanded further between days 7 to 14 p.i. to become >75% of total liver CD8 T cells and gradually and simultaneously lost the expression of CD62L between days 10 to 23 p.i., indicating their ability to reside in the nonlymphoid peripheral tissue like the liver as CD8 T_RM_ cells (Fig 1G) [32].

### RHV-specific T cells target multiple viral proteins

**Fig 2:**
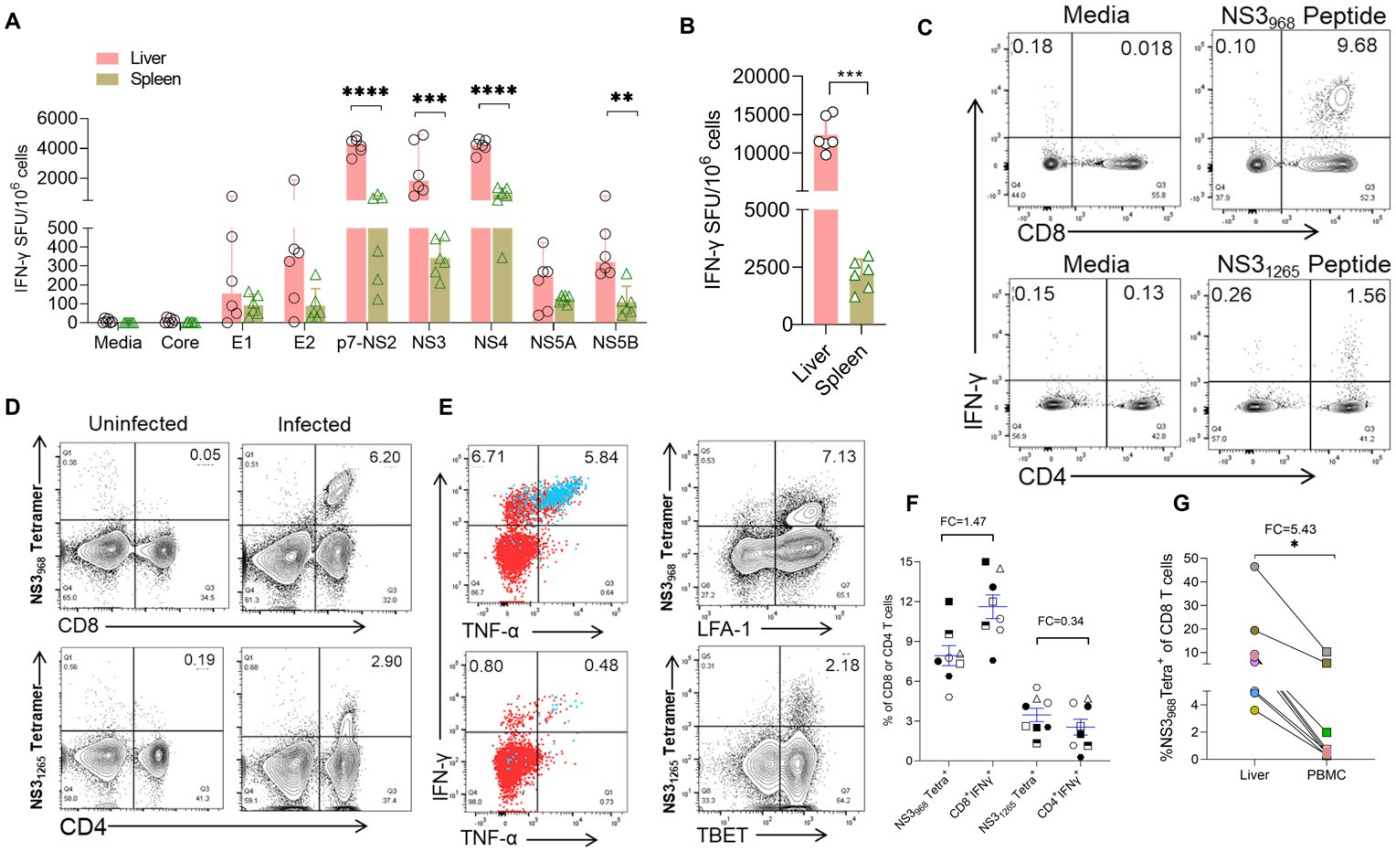
Specificities of RHV-specific T cells and construction of MHC class I and II tetramers. **(A)** RHV infected mice were euthanized on day 17 p.i. to analyze virus-specific T cell responses targeting different RHV proteins in livers (red bars) and spleens (green bars) using IFN-γ ELISPOT assays. Mononuclear cells were stimulated with viral peptides representing different structural and nonstructural proteins (2 μg/mL) for 40–48 h. Each data represents cells pooled from the livers or spleens of 2–3 mice. SFU is spot-forming units. **(B)** Comparison of total RHV-specific T cells in the livers and spleens of infected mice. **(C)** ICS analysis to confirm the specificities of selected immunodominant class I and class II epitopes identified in the NS3 protein of RHV. Flow plots show the percentage of CD8 and CD4 T cells producing IFN-γ following 5-hrs *ex vivo* stimulation with the selected peptides (5 μg/ml). **(D)** Representative flow plots show the frequencies of intrahepatic CD8 and CD4 T cells that specifically bind the NS3_968_ MHC class I (*H-2D*^*b*^) and NS3_1265_ MHC class II (*H-2A*^*b*^ or *I-A*^*b*^) tetramers. **(E)** Intracellular cytokine [IFN-γ, TNF-α, and IL-2 (as blue color)] and expression of LFA-1, and transcription factor (T-bet) staining of intrahepatic T cells following 5-h stimulation with the NS3_968_ or NS3_1265_ peptides. **(F)** Comparison of NS3_968_ or NS3_1265_ tetramer labeled T cells and proportion of total T cells producing IFN-γ after *ex vivo* stimulation with NS3_968_ or NS3_1265_ peptides of individual mice (n = 8). **(G).** Relative frequencies of NS3_968_ tetramer-specific CD8 T cells in the liver and PBMC of nine individual RHV infected mice. All statistical analyses used a two-tailed unpaired t-test (F-G, paired t-test), and only significant p-values are shown, where p-values <0.05 (*), <0.01 (**), <0.001 (***), and <0.0001 (****). Fold changes were calculated using means ± standard errors of the means [SEM].

T cells play an important role in the clearance and control of many viral infections, including HCV and RHV [22,27,33]. We reported that RHV clearance in lab mice is T cell-dependent since selective depletion of CD4 T cells before infection resulted in chronic RHV infection, and transient depletion of CD8 T cells delayed RHV clearance [27]. Here, we analyzed the nature and breadth of RHV-specific T cells in C57BL/6J lab mice. We first assayed T cells isolated from the livers and spleens of infected mice against 11 pools of 18 amino acid long peptides covering the entire RHV polyprotein, as described in our earlier studies on the RHV-rat model [22,26,28]. RHV-specific T cells, identified as T cells producing IFN-γ after *ex vivo* stimulation with RHV peptides, were detected against all viral structural and nonstructural (NS) proteins except the core protein (Fig 2A). The frequencies of intrahepatic T cells targeting NS proteins were 4.37-fold higher than those targeting structural proteins after adjusting for the difference in their respective length. Notably, the highest proportions of RHV-specific T cells were specific for the peptides representing the p7+NS2, NS3, and NS4 proteins. Similar dominance of T cells targeting the NS proteins was reported in HCV-infected humans and chimpanzees [30,33,34]. Since, like HCV, RHV also establishes a strictly hepatotropic infection, we sought to compare the frequencies of RHV-specific T cells in leukocytes isolated from the livers and spleens of infected mice. The frequencies of RHV-specific T cells in the liver were, on average, 5.6-fold higher than in the spleen of the same mice (Fig 2B).

### Development of mouse MHC class I and class II tetramers to study RHV-specific T cells

The construction of mouse MHC class I and class II tetramers for RHV epitopes was fundamental for studying virus-specific T cells in this recently developed animal model. Towards this, we identified two peptides from the NS3 coding region that selectively induced cytokine production in either CD8 or CD4 T cells of RHV infected mice (Fig 2C). The peptide representing the RHV NS3_1265-1278_ PPGTPVTPHPNVET induced IFN-γ and TNF-α production in CD4 T cells. Since the MHC haplotype of C57BL6/J mice carries a single MHC class II allele *I-A*^*b*^, we used this peptide to construct a class II tetramer. The other NS3 peptide that selectively induced cytokine production in CD8 T cells was used to identify the minimal epitope (NS3_968-976_ SAIYNGSIC) and MHC restriction and then used to construct MHC class I tetramer using *H2-D*^*b*^ clone. The specificities of both tetramers were confirmed by comparing their ability to bind intrahepatic CD8 or CD4 T cells of infected and uninfected mice (Fig 2D). The class I NS3_968_ tetramer selectively stained 4–14% of intrahepatic CD8 T cells of infected mice while showing little or no binding to either CD4 or CD8 T cells of uninfected mice (Fig 2D). Similarly, the class II NS3_1265-1278_ tetramer selectively labeled 2–6% of intrahepatic CD4 T cells of RHV infected mice while showing little or no labeling of either CD8 or CD4 T cells of uninfected mice. Overall, these results confirmed that these two tetramers were suitable for characterizing RHV-specific T cells in the C57BL/6J mice and in other mice strains sharing this MHC haplotype, like congenic mice or commonly used 129S1/SvImJ mice.

We expected that the clearance of RHV infection in mice would result in the formation of antigen-specific memory T cells. Thus, we used the peptides incorporated in MHC class I and class II tetramers for *ex vivo* stimulation of intrahepatic leukocytes of RHV-infected mice (Fig 2E). The intracellular cytokine staining and flow cytometric analysis showed that RHV-specific CD8 and CD4 T cells produced multiple Th1 cytokines, IFN-γ, TNF-α, and IL-2, upon antigen stimulation (Fig 2E). To determine the fraction of antigen-specific T cells that produced antiviral cytokines, we compared the T cells recognized by the tetramers with the cells producing IFN-γ when stimulated with the corresponding peptide (Fig 2F). While the IFN-γ producing CD4 T cells were 11 to 85% (mean, 34%) of the NS3_1265_ labeled CD4 T cells, the IFN-γ producing CD8 T cells were 106–205% (mean, 146%) of the NS_968_ tetramer labeled CD8 T cells of the same mice (n = 8). The higher frequency of IFN-γ producing CD8 T cells can be explained by the NetMHCpan analysis [35] that predicted that both MHC class I alleles of C57BL/6J mice, *H2-K*^*b*^ and *H2-D*^*b*^, could bind the NS_968_ peptide; thus, the frequencies of CD8 T cells producing cytokines after antigenic stimulation were more than the CD8 T cells visualized by *H2-D*^*b*^ MHC tetramer. Since the studies of HCV T cell immunity in humans primarily use PBMCs, we used the MHC class I NS3_968_ tetramer to compare the frequencies of RHV-specific CD8 T cells in the blood and liver of infected mice. On average, the frequencies of NS3_968_ tetramer-specific CD8 T cells in PBMC were 5.4-fold less compared to the liver of the same mice (Fig 2G).

### The phenotype of RHV-specific effector and memory T cells

**Fig 3:**
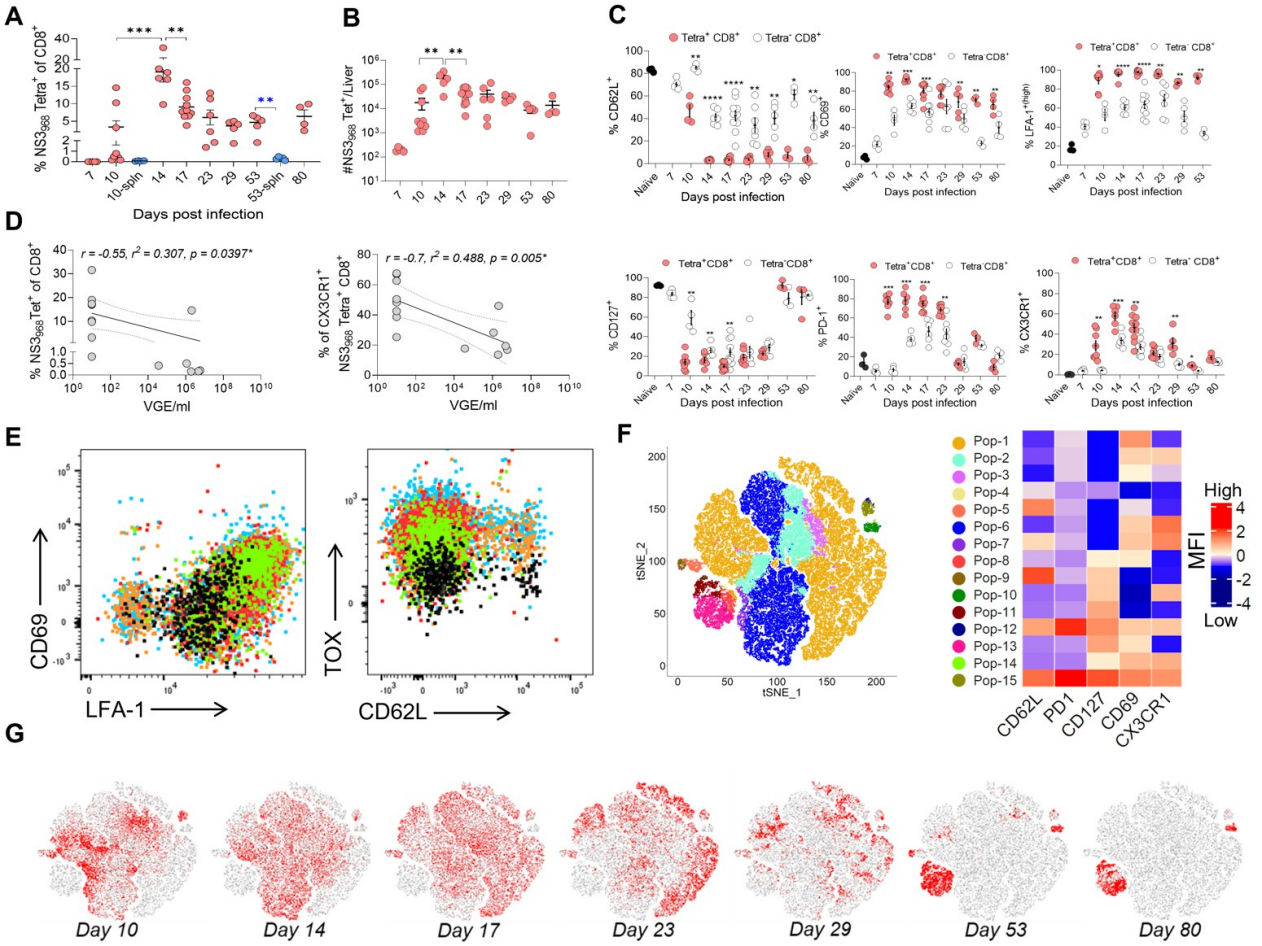
Serial analysis of RHV-specific CD8 T cells in the liver. **(A)** The frequencies of NS3_968_-specific CD8 T cells in the liver (red) and spleen (blue) of RHV infected mice. Asterisks show statistically significant differences. Blue asterisks depict the differences between frequencies of Tet+ cells in the liver and spleen. **(B)** The total number of NS3_968_-specific CD8 T cells in the liver after RHV infection. **(C)** Expression of the surface markers of T cell activation, tissue residency, and memory on NS3_968_-specific CD8 T cells and NS3_968_-negative CD8 T cells in the liver of RHV infected mice. The baseline expression values of these markers in naïve mice are shown as black circles for comparison. The x-axis displays the % of cells expressing the T cell markers on the NS3_968_- specific (filled red circle) or NS3_968_-negative CD8 T cells (empty circle). Each data represents 1–2 mice, and >6 mice were analyzed in >2 independent experiments for each time point. Statistical analyses to compare the expression of T cell marker on tetramer+ and tetramer- CD8 T cells were done using a two-tailed paired t-test, and only significant p-values are shown, where p-value <0.05 (*), <0.01 (**), <0.001 (***), and <0.0001 (****). **(D)** Simple linear regression analysis between the titers of RHV viremia on days 10 and 14 p.i. and NS3_968_-specific CD8 T cells (left panel) and the CX3CR1^+^ NS3_968_-specific CD8 T cells (right panel) in corresponding mice (n = 14). **(E).** Coexpression of T cell markers of tissue-residency, CD69 and LFA-1, and CD62L and TOX, on NS3_968_-specific CD8 T cells from livers of 4 mice (displayed as different colors), and from spleens of the same mice (displayed as black) on day 17 p.i. **(F and G)**. tSNE analysis shows the diversity and changes in the phenotype of NS3_968_-specific CD8 T cells during RHV infection and clearance. FlowSOM was used to cluster NS3_968_-specific CD8 T cells into 15 subpopulations based on the relative expression of different T cell markers. tSNE plots show the expression of T cell markers on different subpopulations **(F)** and the dynamics of these markers in these populations over time **(G)**. The tSNE analysis was done by pooling the data of liver-infiltrating NS3_968_-specific CD8 T cells of 4–6 mice for each time point.

Next, we used RHV NS3_968_ and NS3_1265_ tetramers to characterize the nature of virus-specific T cells in the livers and spleens during and after the clearance of RHV infection. NS3_968_-specific CD8 T cells significantly increased in the liver between days 7 to 14 after infection (Fig 3A). Although NS_968_-specific CD8 T cells were also detected in spleens on day 10 p.i., their frequencies were >10-fold lower than those in the livers of the same mice. The highest frequencies of NS3_968_-specific CD8 T cells were observed on day 14 p.i., representing 10–35% of the total liver CD8 T cells. After day 14 p.i., the frequencies of NS3_968_-specific CD8 T cells declined and stabilized after day 23 p.i., representing 4–5% of the liver CD8 T cells until day 80 p.i. (Fig 3A). NS3_968_-specific CD8 T cells were also detected in the spleens on day 53 p.i. but their frequencies were ~10-fold lower than in the livers. The expansion of NS3_968_-specific CD8 T cells in the liver was also evident from the significant increase in their absolute numbers from day 7 to 14 p.i. (Fig 3B). Notably, the frequency of NS3_968_-specific CD8 T cells and the CX3CR1 expressing NS3_968_-specific CD8 T cells correlated inversely with RHV viremia between days 10 to 14 p.i (Fig 3D). To determine the serial changes in RHV-specific CD8 T cells, we compared the expression of T cell differentiation markers on NS3_968_-specific CD8 T cells and other liver-infiltrating CD8 T cells at serial time points after infection (Fig 3C). While 30–50% of NS_968_ specific CD8 T cells were CD62L^+^ at 10 days p.i., >95% of these cells became CD62L^-^ before day 14 p.i.. However, almost all NS3_968_-specific CD8 T cells were CD62L^-^ after virus clearance and until the end of the study at 80 days p.i. Interestingly, expression of CD69 and LFA-1, the two primary markers of tissue (liver)-resident T cells, remained high on 60–95% of all liver-infiltrating NS3_968_ specific CD8^+^ T cells between days 10 to 80 p.i. Additionally, the expression of CD127, the alpha chain of the interleukin-7 receptor, was high on naïve CD8 T cells but lower on NS3_968_-specific CD8 T cells from days 10 to 29 p.i. during the effector phase of the immune response. Subsequently, 80–100% of NS3_968_-specific CD8 T cells expressed CD127 at days 53 and 80 p.i., indicating the formation of long-lived memory T cells [36]. The NS3_968_-specific CD8 T cells upregulated PD-1 expression between days 10 to 23 p.i. but subsequently downregulated the PD-1 expression between days 29 to 80 p.i (Fig 3C). Although PD-1 as a T cell inhibitory receptor plays a significant role in CD8 T cell exhaustion during chronic infections, PD-1 expression also increases during the early phase of T cell activation [37]. Since the expression of the chemokine receptor CX3CR1 discriminates memory CD8 T cells with cytotoxic effector function from those with proliferative potential in humans and mice [38], we assessed serial changes in CX3CR1 expression on NS3_968_-specific CD8 T cells. The frequencies of CX3CR1^+^ NS3_968_ specific CD8 T cells inversely correlated with RHV viremia during the acute phase, followed by a rapid decline in frequencies of these cells from day 17 to 23 p.i. (Fig 3D).

Next, we compared the nature of NS3_968_-specific CD8 T cells in the livers and spleens of mice at day 53 p.i. The virus-specific CD8 T cells in livers showed higher expression of CD69 and LFA-1 while showing lower expression of CD62L compared to the cells in spleens (Fig 3E). As reported earlier, the liver-resident memory CD8 T cells lack expression of CD103, an essential integrin for T cell residence in epithelial tissues, but upregulate the adhesion molecule LFA-1 that interacts with ICAM-1 and allows CD8 T cells to patrol the liver sinusoids [32]. Interestingly, the liver infiltrating NS3_968_-specific CD8 T cells expressed high levels of thymocyte selection–associated high-mobility group box (TOX) protein compared to the NS_968_-specific CD8 T cells in the spleen. However, TOX expression was also shown to reflect the activation of T cells and does not necessarily correlate with T cell dysfunction [39,40]. Altogether, RHV infection induced robust expansion of virus-specific CD8 T cells with cytotoxic effector function in the liver. The virus-specific CD8 T cells contracted but survived as CD8 T_RM_ cells in the liver after virus clearance, as evident from their coexpression of LFA-1 and CD69 and lack of expression of CD62L.

To further examine how the phenotype of NS3_968_-specific CD8 T cells changed during infection and clearance of RHV, we performed a *t*-distributed stochastic neighbor embedding (*t*-SNE) analysis of these cells from days 10 to 80 p.i. (Fig 3F and 3G) The NS3_968_-specific CD8 T cell population comprised more heterogeneous subpopulations during the effector phase on days 10 and 14 p.i., compared to the memory phase on days 53 and 80 p.i. However, even the less heterogeneous populations of NS3_968_-specific CD8 T cells observed during day 80 p.i. were made of at least three distinct subpopulations, P-10 (CD62L^-^, PD-1^low^, CD127^+^, CD69^-^, and CX3CR1^+^), P11 (CD62L^-^, PD-1^low^, CD127^+^, CD69^-^, and CX3CR1^-^), and P13 (CD62L^-^, PD-1^low^, CD127^+^, CD69^+^, and CX3CR1^-^). Among these, the P13 was the majority population on day 80 p.i. Importantly, low expression of PD-1 and high expression of CD127 on all three of these NS_968_ specific CD8 T cell subpopulations, together with coexpression of CX3CR1 in one subpopulation on day 80 p.i. indicate that acute clearance of RHV infection resulted in the formation of liver CD8 T_RM_ cells capable of self-renewal and cytotoxic activity [36,38].

**Fig 4:**
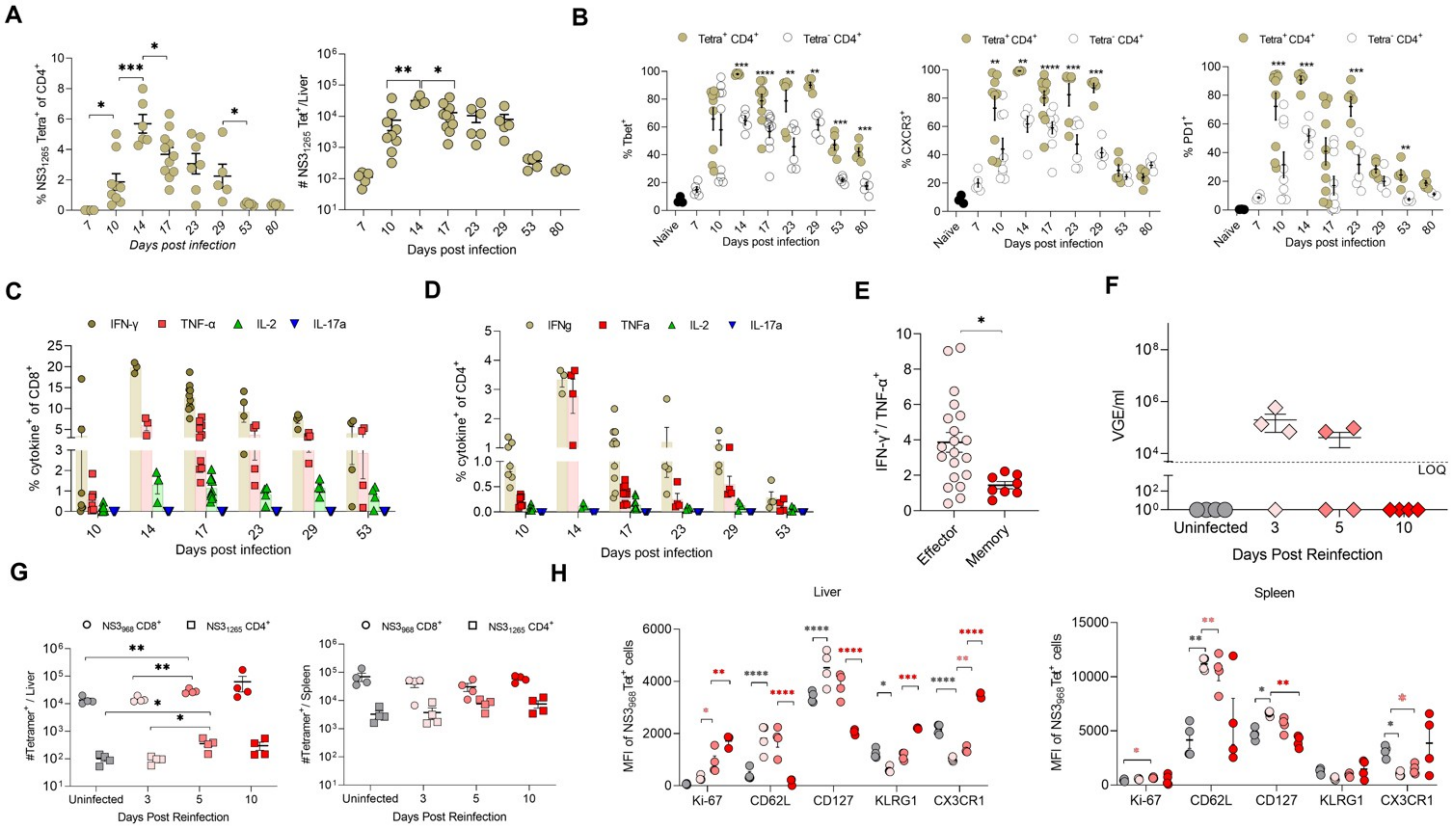
Phenotype and function of RHV-specific T cells and analysis of memory T cell response during RHV reinfection. **(A)** The frequencies and total numbers of NS3_1265_ specific CD4 T cells in the livers of RHV infected mice. **(B)** Kinetics of T-bet, CXCR3, and PD-1 expression on NS3_1265_-specific and non-specific CD4 T cells in the liver of mice. The baseline expression values of these markers in naïve mice are shown as black-filled circles. Each data represents 1–2 mice, and >6 mice were analyzed in >2 independent experiments for each time point. Statistical analyses to compare the expression of T cell marker on Tet^+^ and Tet^-^ CD4 T cells were done using a two-tailed paired t-test, and only significant p-values are shown, where p-value <0.05 (*), <0.01 (**), <0.001 (***), and <0.0001 (****). **(C)** The % of CD8 T cells and **(D)** CD4 T cells producing IFN-γ, TNF-α, IL-2, and IL17 after 5-hrs *ex vivo* stimulation with NS3_968_ and NS3_1265_ peptides at 5μg/ml concentration and analyzed by intracellular cytokine staining. **(E)** The proportions of NS3_968_-specific CD8 T cells producing IFN-γ versus TNF-α during effector (days 10, 14, and 17 p.i.) and memory (days 29 and 53 p.i.) phases. **(F)**. RHV-cleared mice were reinfected >80 days after the first infection with a 10-times higher dose (10^5^ VGE/mice) of mouse-adapted RHV variant to analyze the nature of viremia and recall T cell response on days 3, 5, and 10 p.i. **(G)** RHV reinfection was associated with a significant increase in the total number of NS3_968_-specific CD8 T cells and NS3_1265_-specific CD4 T cells in the liver but not in the spleen. **(H)** Changes in the NS3_968_-specific CD8 T cells in the liver and spleen of mice after RHV reinfection. Black, light red, red, and intense-red circles depict the NS3_968_-specific CD8 T cells for uninfected, day 3, day 5, and day 10 time points, respectively. Asterisks show statistically significant differences: black for uninfected vs. day 3 p.i., light red for day 3 vs. day 5 p.i., and red for day 5 vs. day 10 p.i. Statistical analyses were done using a two-tailed unpaired t-test, and only significant p-values are shown, where p-value <0.05 (*), <0.01 (**), <0.001 (***), and <0.0001 (****).

The frequencies and total numbers of NS3_1265_ tetramer-specific CD4 T cells also increased in the liver between days 10 to 29 p.i., followed by their contraction to form <1% of the liver CD4 T cells between days 53 to 80 p.i. (Fig 4A). Most NS3_1265_-specific CD4 T cells co-expressed T-bet and CXCR3, indicating their Th1 skewing (Fig 4B). Moreover, most of these cells were PD-1^+^ between day 10–23 p.i., but then the PD-1 expression declined, and only ~25% of these cells expressed low levels of PD-1 during the memory phase on days 53 and 80 p.i. Similarly, only ~25% of these cells expressed T-bet or CXCR3 during the memory phase. Finally, to determine the cytokine-producing ability of RHV-specific T cells, we stimulated the intrahepatic leukocytes *ex vivo* using class I and class II peptides incorporated in the tetramers, followed by an intracellular cytokine staining. The RHV-specific CD8 and CD4 T cells primarily produced IFN-γ, TNF-α, and IL-2 during RHV infection and after virus clearance between days 10 to 80 p.i. (Fig 4C and 4D). We also assayed the production of IL-4, IL-10, IL-15, IL-17, and IL-21 on days 14, 21, and 53 p.i. but no significant increases in the expression of these cytokines were observed. Over time, the changes in the frequencies of cytokine producers positively correlated with the changes in the frequency of class I and class II tetramer-specific cells. However, the proportions of virus-specific CD8 T cells making only IFN-γ were higher during the effector phase than in the memory phase (Fig 4E).

### Liver resident memory CD8 T cells respond to RHV reinfection

We reported that the RHV-cleared mice develop CD8 T cell-dependent protection against reinfection [27]. To characterize the nature of recall T cell response, we reinfected RHV-cleared mice 80–100 days after clearance and compared the changes in the phenotype of the virus-specific T cells in the liver and spleen. The reinfected mice had low-titer viremia on days 3 and 5 p.i. and cleared the viremia by day 10 p.i., indicating the lack of sterilizing immunity (Fig 4F). Analysis of total NS3_968_-specific CD8 T cells and NS3_1265_ tetramer-specific CD4 T cells in the liver and spleen of reinfected mice showed a significant increase in the absolute numbers of virus-specific CD8 and CD4 T cells in the liver but not in the spleen between days 3 to 10 p.i. (Fig 4G). Next, we analyzed the changes in NS3_968_-specific CD8 T cells in the liver and spleen of mice after reinfection. The expression of Ki67, KLRG-1, and CX3CR1 significantly increased between day 3 to 10 p.i. on the NS3_968_-specific CD8 T cells in the liver but not in the spleen, indicating that the control of RHV reinfection was associated with the proliferation of liver-resident NS3_968_-specific CD8 T cells with effector and cytotoxic functions. Moreover, there was a significant increase in the frequencies of CD62L^+^ NS_968_-specific CD8 T cells in the spleen on days 3 and 5 p.i. indicating a simultaneous expansion of central memory cells upon reinfection. A similar serial comparison of NS3_1265_ tetramer-specific CD4 T cells showed no significant changes in their phenotype after RHV reinfection, indicating that the memory CD8 T cells play a dominant role in the clearance of RHV reinfection. These results supported our previous data that the in vivo depletion of CD8 T cells before reinfection can delay RHV clearance, and the depletion of CD4 T cells before reinfection did not affect the rapid clearance of secondary RHV infection in the mouse model [27].

### Chronic RHV infection leads to T cell exhaustion and the development of severe liver diseases

**Fig 5:**
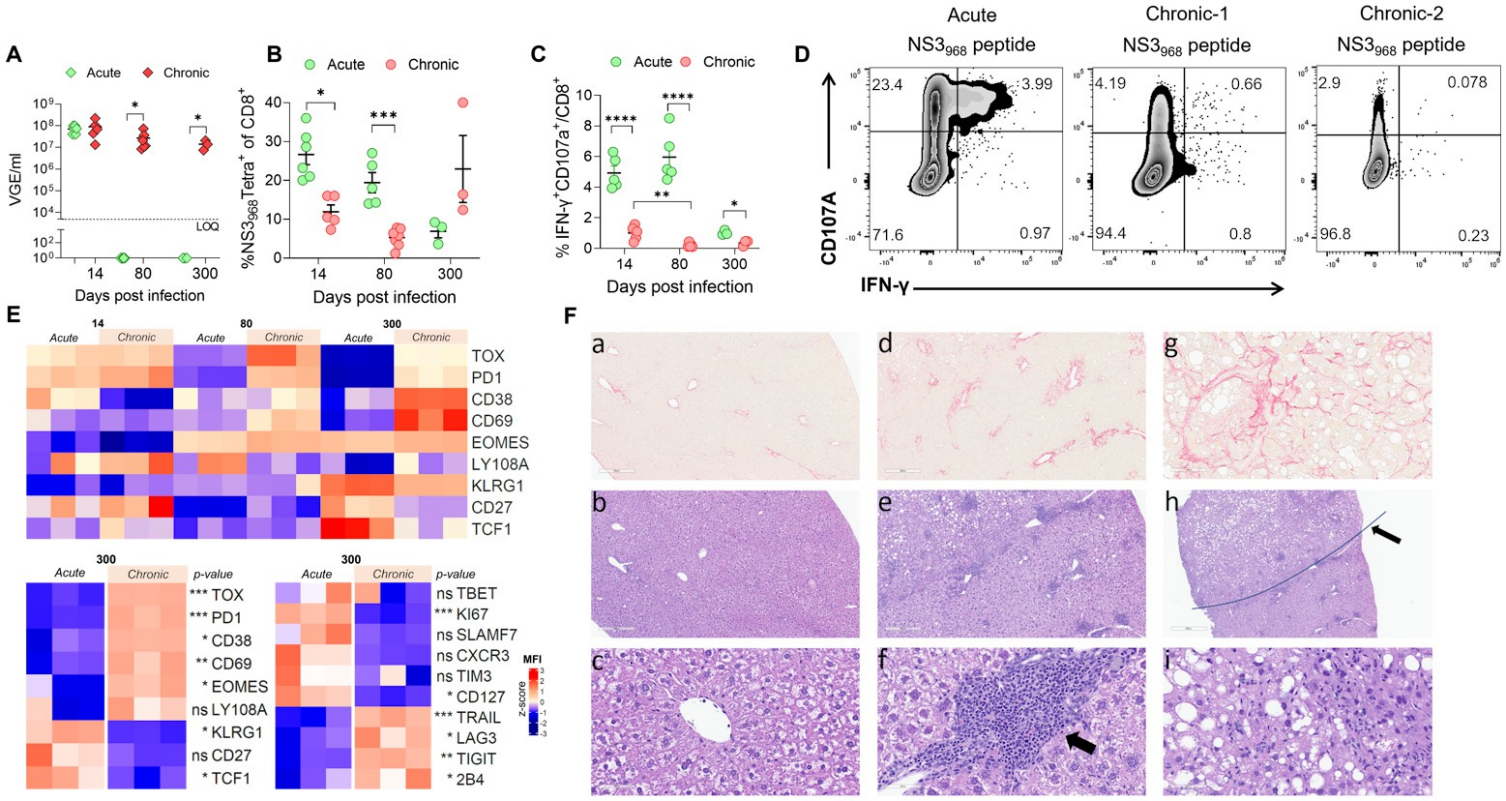
Chronic RHV infection leads to T cell exhaustion and the development of severe liver diseases. **(A)** RHV viremia in undepleted (acute) and CD4-depleted (chronic) mice. **(B)** The frequencies of NS3_968_-specific CD8 T cells in mice with acute and chronic infection. **(C)** ICS analysis of liver-infiltrating CD8 T cells stimulated *ex vivo* using NS3_968_ peptide indicates that the NS3_968_-specific CD8 T cells in chronically infected mice are exhausted since they fail to produce IFN-γ or express degranulation marker CD107A during the early and late phases of infection. **(D)** Representative flow plots showing the exhausted nature of CD8 T cells in acute and two chronic mice infected for 80 days p.i. **(E).** Comparison of cell surface markers and transcription factors on NS3_968_-specific CD8 T cells on day 14, 80 and 300 p.i. in mice with acute and chronic infection. A more extensive flow cytometry panel compared the NS3_968_-specific CD8 T cells in cleared and chronic mice on day 300 p.i. (lower panels). Statistical analyses were done using a two-tailed unpaired t-test, NS means nonsignificant and significant p-values are shown, where p-value <0.05 (*), <0.01 (**), <0.001 (***), and <0.0001 (****). **(F)**. Comparative liver histology of mice with acute and chronic RHV infection (day 300 p.i.). Cleared mouse (a, b, c). (a) Normal portal tracts outlined by collagen (red) and are regularly distributed (Picrosirius Red). (b) Low magnification view of normal-appearing parenchyma (H&E). (c) Higher magnification of hepatocytes with normal age-related hepatocyte variation and clear cytoplasmic spaces due to glycogen accumulation. Chronically infected mouse: (d, g) Increased fibrosis emanating from portal tracts with evidence of bridging and sinusoidal fibrosis. The tumor-bearing liver at the top left corner lacks portal tracks and has lipid vacuoles. (e, h) A hepatocellular carcinoma at the top of the image has a irregular border (line) with the non-neoplastic but inflamed liver with regularly spaced portal tracts below. (f) Higher magnification of lymphocytic infiltrate in the portal region. (i) Higher magnification of the hepatocellular carcinoma with loss of sinusoidal architecture, prominent pleomorphism of hepatocytes, and lipid vacuolation.

Transient depletion of CD4 T cells during early RHV infection resulted in delayed clearance or chronic infection in most mice (Fig 5A). To determine the nature and fate of virus-specific CD8 T cells during chronic RHV infection, we compared the phenotype and function of antigen-specific CD8 T cells in mice with cleared or chronic infection on days 14, 80–100, and 300 p.i. On day 14 p.i., the frequencies of NS3_968_ Tet^+^ CD8 T cells in CD4 T cells depleted mice (chronic) were significantly lower compared to the undepleted mice (acute), indicating a significant decrease in their expansion in the liver in the absence of CD4 T cells (Fig 5B). The frequencies of NS3_968_-specific CD8 T cells declined further during the early chronic phase (day 80–100 p.i.) but after that increased during the late chronic phase (day 300 p.i.). ICS analysis revealed that the NS3_968_-specific CD8 T cells in chronic mice were significantly impaired in their ability to produce IFN-γ (Fig 5C) during both the early and late phases of chronic infection. Notably, the NS3_968_-specific CD8 T cells represented 10–40% of the total liver-resident CD8 T cells, despite the persistent high-titer viremia for 300 days, retained their ability to degranulate (CD107A expression), and expanded during the late chronic phase (Fig 5B–5D). Altogether, these results showed that the RHV-specific CD8 T cells continued proliferation despite the persistent exposure to high-titers of antigen for months and largely resembled the exhausted CD8 T cells (Tex) characterized during chronic LCMV cl-13 infection in mice [41,42].

Next, we compared the phenotype of NS3_968_ Tet^+^ CD8 T cells in mice with acute and chronic infection (Fig 5E). On day 300 p.i., compared to the cleared mice, the NS3_968_ Tet^+^ CD8 T cells in the liver chronic mice had significantly higher expression of TOX, PD-1, CD38, CD69, EOMES, TRAIL, LAG3, TIGIT, and 2B4, while significantly lower expression of KLRG1, TCF1, Ki67, and CD127. Serial analysis of the NS3_968_ Tet^+^ CD8 T cells in the liver of chronic mice on days 14, 80–100, and 300 p.i. showed a significant gradual increase in the expression of CD38, CD69, EOMES, and KLRG1, while no significant differences were observed in the expression of PD-1 and TOX. Notably, a comparison of NS3_968_ Tet^+^ CD8 T cells in the liver during the early and late phases of chronic infection showed a significant decrease in the expression of TOX and PD-1 while a significant increase in the expression of CD38, CD69, and KLRG1 during the late phase (Fig 5E).

Since RHV infection in lab mice and rats induced liver inflammation and diseases [23,27], we compared the histology of the liver in mice with cleared and chronic RHV infection on day 300 p.i. (Fig 5F). The H&E-stained liver sections of all three chronic mice showed the presence of lymphocytic infiltrates in the portal region, loss of sinusoidal architecture, prominent pleomorphism of hepatocytes, and lipid vacuolation. In two of the three mice, we also observed hepatocellular carcinoma with a distinct border with the non-neoplastic but inflamed liver with regularly spaced portal tracts (Fig 5F). To determine the extent of fibrosis, we used Picrosirius Red staining. We determined that all three chronic mice had grade III fibrosis emanating from portal tracts with evidence of bridging and extending along sinusoids. In contrast, the cleared mice showed normal-appearing parenchyma, regularly distributed and normal portal tracts outlined by collagen, no evidence of portal inflammation, and only normal age-related hepatocyte variation and clear spaces due to glycogen (Fig 5F).

### The unique phenotype and transcriptome of RHV-specific CD8 T_RM_ cells

**Fig 6:**
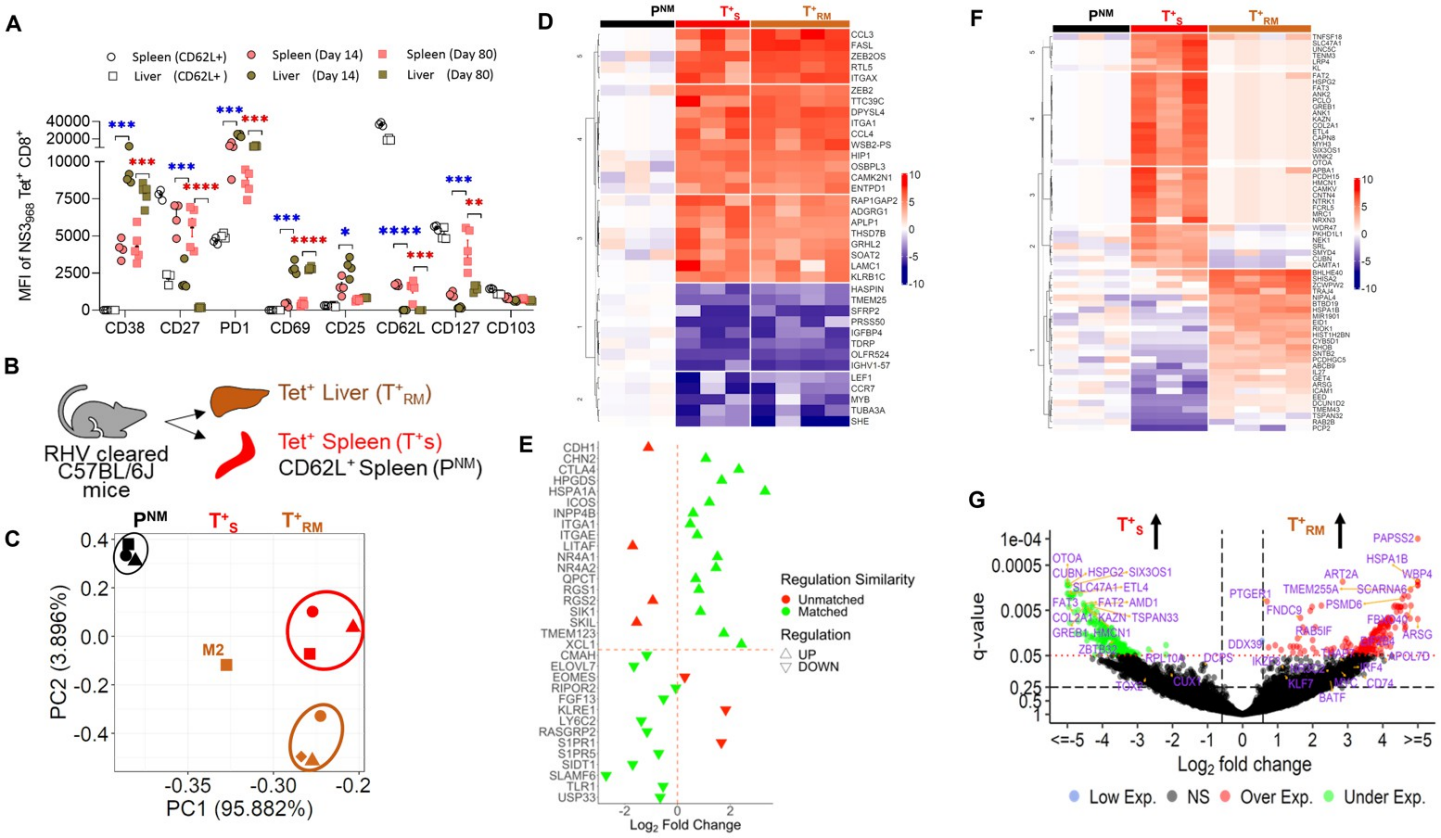
The phenotype and transcriptome of RHV-specific liver CD8 T_RM_ cells. **(A).** Comparative expression of cell surface markers on the NS3_968_-specific CD8 T cells from the liver (T^+^_RM_) and spleen (T^+^_S_) of RHV cleared mice. Asterisks show statistically significant differences between the expression of T cell markers on NS3_968_-specific CD8 T cells in the liver and spleen, where blue asterisks depict the differences between day 14 p.i. and red asterisks depict the differences between day 80 p.i. **(B)** NS3_968_-specific CD8 T cells from the liver (T^+^_RM_) and spleen (T^+^_S_) of RHV cleared mice, and CD62L^+^ CD8 T cells (P^NM^) from the spleen of naïve mice were labeled and sorted using BD Influx Cell Sorter to prepare RNA-seq libraries. **(C)**. PCA analysis of the normalized read counts for all samples. **(D).** Pooled data of all T^+^_RM_ and T^+^_S_ samples were compared to identify the 100 most over- and under-expressed DEGs. Thirty-six DEGs that showed consistent log2 fold change gene expression patterns between all samples of the groups are shown together with P^NM^ samples. **(E).** The heatmap shows log2 fold change expression pattern of DEGs between RHV-specific T^+^_RM_ and T^+^_S_ analyzed in this study compared to the pattern of 33 core-signature DEGs reported for CD8 T^+^_RM_ cells of skin, lung, gut, and liver. **(F).** The heat map shows log2 fold change expression pattern of 63 DEGs that were the most up- or down-regulated between T^+^_RM_ and T^+^_S_ of RHV-cleared mice. **(G).** The volcano plot shows selected significantly differentially expressed genes including T cell transcription factors between T^+^_RM_ and T^+^_S_ cells.

Since RHV infects and replicates only in the hepatocytes and offers a unique model to study hepatotropic viral infection, we aimed to define the phenotype and transcriptome of NS3_968_-specific effector and memory CD8 T cells residing in the liver and spleen of mice. The liver infiltrating NS3_968_-specific CD8 T effector cells on day 14 expressed significantly higher levels of CD25, CD38, CD69, and PD-1, and lower levels of CD27, CD62L, and CD127, compared to their spleen homologs (Fig 6A). Similarly, the long-lived memory CD8 T cells in spleen and liver differed remarkably in their phenotype. Overall, the virus-specific NS3_968_-specific CD8 T_RM_ cells had significantly higher expression of CD38, CD69, and PD-1 compared to their homologs in the spleen. Notably, there was no significant difference in the expression of CD103, a known marker of tissue residency, between the liver and spleen cells (Fig 6A).

Next, we aimed to characterize the transcriptome of NS3_968_-specific long-lived memory CD8 T cells residing in the liver and spleen of mice >2 months after virus clearance. For comparison, we chose CD62L^+^ CD8 T cells of the spleen to obtain baseline gene expression profiles of the pool of naïve and central memory (P^NM^) CD8 T cells (Fig 6B). The principal component analysis showed that the samples belonging to different groups clustered separately, and the NS3_968_-specific memory CD8 T cells, irrespective of their source organ, liver, or spleen, were transcriptionally distinct from the CD62L^+^ CD8 T cells (Fig 6C). Further analysis using Pearson’s correlation matrix showed that NS3_968_-specific liver CD8 T_RM_ cells from one of the four mice had a weaker correlation with others in the group (Fig 6C); however, we did not exclude this sample from further analysis and considered it as a biological variable. The RHV-specific memory CD8 T cells, irrespective of their origin, had significantly different gene expression profiles compared to the spleen CD62L^+^ CD8 T cells (Fig 6D). Using Ingenuity Pathway Analysis [43], we observed that RHV-specific CD8 T_RM_ cells had upregulation of differentially expressed genes (DEGs) associated with activation of cytotoxic T cells and cytokine signaling including CCL3, CCL4, IL2RA, NRP2, CCL5, CD40, APOE, SOX9, C3, IFNG, CASP1, IGF1, CSF2RB, IL17RD, RORA, TNFSF10, FOS, FOSB, IL1RL1, RUNX2, TNFRSF1B, and ITGB2. The downregulated DEGs included CCR7, SELL (CD62L), SELE (CD62E), IGF1R, TYMP, AR, SREBF1, and CSF1R. The RHV-specific memory CD8 T cells in the liver and spleen also had upregulation of DEGs associated with IL13, IL15, integrin, TREM1, and FAK signaling and downregulation of DEGs associated with E1F2, RHOGDI, and HIPPO signaling. The upstream regulators shared between the RHV-specific memory CD8 T cells in the liver and spleen included IL1, IL2, IL12, IL15, IL21, IFNG, CSF2, TGFB1, and KLRK1.

Next, we identified the DEGs signature of the RHV-specific liver T_RM_ CD8 T cells. First, we compared the pattern of DEGs identified in RHV-specific T_RM_ CD8 T cells with the core signature genes identified in the skin, gut, and lung CD8 T_RM_ cells [44]. We found 79% concordance in the expression pattern of core signature genes in the RHV-specific CD8 T_RM_ cells (Fig 6E). Our results also align with the gene expression pattern of the liver T_RM_ CD8 T cells induced by the malaria vaccine [45]. Interestingly, our unbiased analysis identified 397 genes that were significantly (adjusted p-value or q-value <0.05) differentially expressed in the RHV-specific T_RM_ CD8 T cells compared to the RHV-specific CD8 T cells from the spleen (Fig 6G). Of these 397 DEGs, 63 genes showing consistent upregulation or downregulation in at least 3 of the 4 T_RM_ samples are shown in Fig 6F. We also compared the differences in expression of T cell transcription factors [46,47]. We identified ten transcription factor genes that were significantly differentially expressed in the T_RM_ cells [p-value< = 0.05, adjust p-value < = 0.25] (Fig 6G). Among these, the overexpression of IRF4 and BATF in RHV-specific CD8 T_RM_ cells is notable since the maintenance of tissue-resident memory T cells appears to depend on IRF4 expression [47,48]. Analysis of gene expression pattern suggested that the upstream regulators of RHV-specific liver T_RM_ CD8 T cells with significant activation included TNFSF13B, CREB1, KLF6, TCR complex, IL3, RUNX, CD40, IL2, IL12A, and TNF, and with significant inhibition was predicted for SIRT1, HNF1A, MAP2K1, KLF4, TEAD2, MAP3K7, FOSL1, and SOX2. The top upregulated genes in RHV-specific T_RM_ CD8 T cells were *HSPA1A*, *TMED10*, *PSMD6*, *USP42*, *IL27*, *TRAJ4*, *ICAM1*, *GZMB*, *H2-M3*, *TCF7L1*, *CCL17*, *IRF2BP1*, and *IL12RB1*. Our data broadly align with the transcriptional profiles of human lung T_RM_ cells [47].

Finally, since the RHV-specific CD8 T cells from the spleen were all CD62L negative and thus might not be considered the prototypical resident of a lymphoid organ or central memory T cells, we compared the liver-T_RM_ cells with the CD62L^high^ CD8 T cells of the spleen. The CD62L^high^ subsets could provide a baseline transcriptome of central memory and naïve CD8^+^ T cells in the spleen. The top overexpressed DEGs in T_RM_ cells included *CCL3*, *ITGAX*, *GZMB*, *ITGA1* (CD49), *CCL4*, *CISH*, *IFNG*, *TRAJ4*, *LGALS2*, *NR4A2*, *LAG3*, *CCL5*, *TBX21* (TCF-1), *SLAMF1*, *CTLA4*, and *PDCD1*. The top underexpressed DEGs in T_RM_ cells included *SHE*, *SELE*, *SLC16A5*, *IGFBP4*, *SELL* (CD62L), *USP28*, *TLR1*, *CCR7*, *MYB*, *TREML2*, *TREM25*, *SLAMF6*, *IGF1R*, *FBXO3*, *IFNGR2*, *CEACAM1*, *LEF1*, *PECAM1*, *STX2*, *CXCL17*, *IL6ST*, *CD163*, *XCL1*, and *KLRC2*. Notably, the RNA-seq data corroborated the protein expression observed by immunostaining and flow cytometry since the T_RM_ CD8 T cells had significantly lower expression of genes expressing CD62L (selectin-L or SELL) and CCR7.

## Discussion

During the early acute phase of RHV infection in mice, the cellular and immunological changes were more pronounced in the liver compared to the spleen, exemplified by almost 10-fold increase in CD8 T cells in the liver (Fig 1D). The robust expansion of CD8 T cells in the liver was anticipated since hepacivirus replicate exclusively in hepatocytes and thus viral proteins on MHC class I alleles are predominantly presented in the liver, facilitating the on-site clonal expansion of virus-specific CD8 T cells. The expanded CD8 T cells coexpressed CD69 and LFA-1 to enable their retention in the liver [32] and were also positive for CD62L on day 10 p.i., suggesting their recent migration from lymphoid tissue (Fig 1G) [13]. While serial changes in the spleen T cell compartment during acute HCV infection remain unstudied, analysis of sequential liver biopsies from experimentally HCV-infected chimpanzees showed that virus-specific cytolytic T cells could be detected in the liver as early as 2–5 weeks after infection, and their expansion coincides with viral clearance [30,49]. Similarly, the intrahepatic CD8 T cells expanded early after RHV infection in mice, and their expansion continued until a week after virus clearance (Fig 1D and 1E). It is plausible that despite the absence of viremia, low-level virus replication or antigen expression in the hepatocytes continues for a few more days.

Since T cells play an essential role in the clearance of HCV infection and reinfection [19,33], the development of mouse MHC class I and II tetramers was critical for studies of virus-specific T cell immunity in the RHV-mouse model. The T cells in RHV-infected mice showed specificities toward multiple viral proteins (Fig 2A). Similar to the T cell responses observed during HCV infection [18], the RHV-specific T cells predominantly targeted the nonstructural proteins, and the frequencies of virus-specific T cells were higher in the liver compared to the spleen during infection and after virus clearance (Fig 3A). The development of *H2-D*^*b*^ and *I-A*^*b*^ based tetramers allowed direct visualization of RHV-specific CD8 and CD4 T cells and analysis of their kinetics and phenotype in the liver and spleen (Figs 3 and 4). Although RHV-specific CD8 T cells can be detected as soon as days 7–10 p.i., they primarily expanded between days 10 to 14 p.i. and started contracting after virus clearance. Notably, the CD8 T cells specific for a single NS3 epitope constituted 5–20% of intrahepatic CD8 T cells even >3 months after virus clearance, indicating the formation of long-lived CD8 T_RM_ cells (Fig 3A). An inverse correlation existed between the frequencies of NS3_968_-specific CD8 T cells expressing CX3CR1 and RHV viremia during the acute phase, between days 10–14 p.i. Notably, the fractalkine receptor CX3CR1 expression discriminates memory CD8 T cells with cytotoxic effector function from those with proliferative potential both in humans and mice [38]. Interestingly, in humans with chronic HCV infection, HCV-specific CD8 T cells expressing CX3CR1 are rare but co-express GzmB and perforin, whereas CX3CR1^-^ HCV-specific CD8 T lack their expression [38]. Thus, it is likely that the differences in the fraction of CD8 T cells expressing CX3CR1 correlate with the fate of hepacivirus infection in mice and humans. Furthermore, the frequency of CD127 expressing HCV-specific T cells but not the expression of exhaustion markers predicts the outcome of acute HCV infection in chimpanzees [50], and the expression of CD127 has been shown to mark activated effector CD8 T cells that are more likely to survive and give rise to robust memory T cell pool [36]. All different subpopulations of the NS3_968_-specific CD8 T_RM_ cells present in the liver after two months of RHV clearance expressed CD127 or IL7R α-chain (Fig 3), indicating their ability to form long-lived memory cells that can survive in the absence of antigen.

To study the formation of RHV-specific CD8 T_RM_ cells, we used tSNE analysis to compare the serial changes in RHV-specific intrahepatic CD8 T cells from day 7 to 80 p.i. Interestingly, the intrahepatic CD8 T cells specific for a single RHV epitope (NS3_968-976_) were heterogeneous and could be divided into several distinct subpopulations based on their expression of T cell markers during the effector and memory phases. Analysis of these subpopulations over time suggested that the dominant subpopulations of RHV-specific intrahepatic CD8 T cells lacking CD127 expression (Pop-1 to 7, Fig 3E) during the early infection contracted after virus clearance. In contrast, the minor subpopulations expressing CD127 (pop-10, 11, and 13) expanded to form the long-lived CD8 T_RM_ cells. Moreover, the dominant subpopulation of RHV-specific CD8 T_RM_ cells expressed CD69 and CD127 and relatively lower levels of PD-1 (pop-13 in Fig 3E) and was negative for CD62L and CX3CR1. Overall, these results align with the observation in the LCMV model, where the T effector cells expressing high levels of CD127 serve as the precursors of the long-lived memory T cells [51]. However, a more accurate analysis of the developmental trajectory of RHV-specific CD8 T_RM_ cells requires high-resolution single-cell RNA-seq analysis.

The RHV-specific effector and memory T cells were robust producers of Th1 cytokines (Fig 4). Comparative analysis of tetramers-specific cells and intracellular cytokine staining indicated that approximately 60–80% of the RHV-specific CD8 T cells could produce IFN-γ, while only 10–15% could produce TNF-α, and only 2–5% could produce IL-2. Interestingly, the antigen-specific CD8 T cells that produced only IFN-γ during the early RHV infection declined after the virus clearance (Fig 4C and 4D). Finally, similar to the other models of viral infections [52], the frequency of virus-specific CD8 IFN-γ-producing cells was substantially higher than the IFN-γ producing CD4 T cells during acute infection and after viral clearance (Fig 4C and 4D).

An interesting observation from our earlier study was that antibody-mediated depletion of CD8 T cells before reinfection resulted in higher viremia titers and delayed virus clearance [27]. To examine how RHV-specific CD8 and CD4 T_RM_ cells respond during reinfection, we characterized the changes in their frequency, phenotype, and function in reinfected mice (Fig 5A–5D). The virus-specific CD8 T_RM_ cells respond rapidly to the reinfection and transition from memory to effector cells, as evident from increased expression of KLRG-1 and decreased expression of CD127. Interestingly, the changes described above in virus-specific CD8 T_RM_ cells were not observed in the spleen immediately after RHV reinfection, suggesting that the T_RM_ cells play a dominant role in the early control and clearance of hepacivirus reinfection (Fig 4G).

During acute infection or vaccination, naive CD8 T cells undergo robust proliferation and clonal expansion to differentiate into effector CD8 T cells that kill target cells and control infections. In contrast, during chronic infection or persistent antigen exposure, the naïve CD8 T cells fail to control or clear the infection or differentiate into memory T cells and attain a state of dysfunction, commonly called T cell exhaustion [41,42]. The spontaneous clearance of RHV in mice depends on the presence of CD4 T cells during the early days of infection [27]. To determine the phenotype and function of RHV-specific CD8 T cells during the chronic RHV infection in mice, we transiently depleted CD4 T cells in mice before RHV infection. The CD4 depleted mice remained chronically infected with high-titer persistent viremia until observed. The RHV-specific CD8 T cells in the liver of these mice expanded during the early phase and showed no significant difference in the expression of PD-1 or TOX but had significantly higher expression of CD27 and low expression of CD38 (Fig 5E). However, their ability to make antiviral cytokines on short-term antigenic stimulation was compromised considerably compared to the cells in undepleted mice, indicating their exhausted state as early as day 14 p.i. (Fig 5B–5D). Furthermore, the functional exhaustion of virus-specific CD8 T cells worsened during the chronic phase, as evidenced by the significant decrease in cytokine-producing CD8 T cells in mice on day 80 or 300 p.i. (Fig 5C). The comparison of virus-specific CD8^+^ T cells of cleared and chronic mice on day 300 p.i. pronounced these differences as evident from significantly higher expression of TOX, PD-1, CD38, CD69, EOMES, TRAIL, LAG3, TIGIT, and 2B4, while significantly lower expression of KLRG1, TCF1, Ki67, and CD127 on the NS3_968_ Tet^+^ CD8 T cells in the liver of chronic mice. However, serial analysis of the NS3_968_ Tet^+^ CD8 T cells in the liver of chronic mice on days 14, 80–100, and 300 p.i. showed a significant gradual increase in the expression of CD38, CD69, EOMES, and KLRG1, while no significant differences were observed in the expression of PD-1 and TOX. These results suggest that chronic RHV infection leads to CD8 T cell exhaustion (Tex), but the virus-specific Tex cells continue to survive and proliferate in the liver during the chronic infection characterized by high-titer persistent viremia.

The observed development of severe liver diseases in mice with chronic RHV infection is highly significant since 15–30% of people with chronic HCV infection remain at risk of developing end-stage liver diseases (Fig 5F). Although only three mice with long-term chronic infection were available for the analysis, the presence of severe liver diseases in all these mice compared to the age-sex matched RHV cleared mice, together with the detection of high-frequencies of virus-specific CD8 T cells in the diseased livers indicate that this model can be used to define the role of virus-specific T cells in hepacivirus immunopathogenesis. However, we emphasize that the frequencies or the nature of virus-specific CD8 T cells or the extent of liver inflammation and diseases in a model of spontaneous chronic hepacivirus infection might remarkably differ from that observed in this study of induced-chronic infection.

The memory CD8 T cells can provide rapid effector functions upon reinfection, but their distribution over different body sites is crucial for maximizing the chance of early pathogen recognition upon reinfection [53]. Comparative analysis of RHV-specific memory T cells during reinfection in the liver and spleen also suggests a rapid transition of RHV-specific CD8 T_RM_ cells to effectors cells, confirming their role in protective immunity. Notably, HCV-specific CD8 T_RM_ cells remain poorly studied due to limited access to the human liver. While transcriptional analysis of liver CD8 T_RM_ cells has been reported, the RHV model provides a unique context where infection of a strictly hepatotropic RNA virus produces long-lived memory cells that predominantly reside in the liver several months after the virus clearance. After confirming that the RHV-specific CD8 T cells from the liver and spleen are phenotypically different (Fig 6A), we determined that the transcriptome of RHV-specific CD8 T_RM_ cells differ significantly from their homologs in the spleen and essentially shares the gene expression pattern previously reported for CD8 T_RM_ cells of skin, lung, gut, and liver (Fig 6C–6G).

The expression of integrins (ITG) that are integral transmembrane glycoproteins and participate in cell adhesion and cell-surface mediated signaling is known to differ between T_RM_ and T_CM_ CD8 T cells. Interestingly, the expression of integrins *ITGA1*(CD49a), *ITGAL* (LFA-1), *ITGAM* (CD11b), and *ITGAX* (CD11C) was significantly higher on RHV-specific CD8 T cells compared to the CD62L^+^ CD8 T cells of the spleen. However, only the expression of *ITGAL*, *ITGA2* (CD49b), and *ITGA10* significantly differed between RHV-specific CD8 cells in the liver and spleen. These observations indicate that the RHV-specific CD8 T cells in the spleen are not as different as T_CM_ and T_RM_ cells observed in previous studies. However, it is plausible that the gene expression levels differ from protein expression levels, and thus, these results require confirmation using protein expression analysis or flow cytometry.

Of the 397 DEGs found between RHV-specific CD8 T_RM_ and spleen cells, the DEGs with the highest expression (reads per million) in T_RM_ cells included *PTGER1*, *DDX39*, *PHYKPL*, *TCTN1*, and *JUNOS*, and those with the lowest expression were *DNAH9*, *ERGIC2*, *SORCS2*, *UBXN7*, and *RUNDC3A*. The DEGs with the highest differential overexpression in T_RM_ cells included *PAPSS2*, *HSPA1B*, *WBP4*, *miR1901*, and *ARSG*, and with the highest underexpression in T_RM_ cells included *HSPG2*, *CUBN*, *SLC47A1*, *OTOA*, and *ETL4*. One of the overexpressed transcripts in T_RM_ cells was *JUN* (C-Jun or AP-1) which was recently identified to prevent exhaustion of activated CD8 T cells, and the overexpression of *JUN* in T cells enhances their expansion potential and functional capacity [54]. Similarly, T_RM_ cell overexpressed *RHOB*, which controls the Rab11-mediated recycling and surface reappearance of LFA-1 in migrating T lymphocytes [55]. Notably, TRM cells overexpressed *IL27*. It has been shown that the CD8 T cell-intrinsic IL-27 signaling safeguards the ability of TCF1^hi^ cells to maintain proliferation and avoid terminal differentiation or programmed cell death. Mechanistically, IL-27 endows rapidly dividing cells with IRF1, a transcription factor that was required for sustained division in a cell-intrinsic manner [56]. Indeed, IRF1 was also overexpressed in the T_RM_ cells compared to the RHV-specific CD8 T cells in the spleen. Among DEGs, significantly underexpressed in T_RM_ cells was *ZBTB32*, whose deficiency in CD8 T cells was shown to enhance virus-specific CD8 T cell responses and increase the formation of virus-specific memory cells [57], CCDC88A, a protein abundantly expressed in lymphoid organs, *FGFR1*, whose expression on T cells helps in their costimulation with TCR, *SMAD1*, *RasGRF2* that mediates T cell signaling, long non-coding mRNA *MEG3*, and *TBX5* that can enhance the expression of NFAT3 to inhibit IL-2 expression. Overall, our results showed that the RHV-specific CD8 T cells in the liver and spleen are traditionally distinct. Interestingly, although RHV-specific CD8 T_RM_ cells share most features of conventional T_RM_ cells described in other contexts [32,44,45,47,58], they were unique in their expression of genes and transcriptional factors for surviving in the liver environment while preserving their ability to mount robust antiviral recall responses.

This study describes the fundamental features of virus-specific T cells in RHV infected mice. The identification of immunodominant T cell epitopes and the newly developed mouse MHC class I and class II tetramers will allow the RHV mouse model to define the liver-specific antiviral response that shapes the nature of liver-resident T cells and the divergent outcomes of hepatotropic infections. Although an inherent limitation of this study is that normal lab mice are not fully susceptible to developing HCV-like spontaneous chronic infection, our results are critical for comparative analyses of T cell dysfunction and immune evasion mechanisms associated with chronic hepacivirus infection in other models and humans. Similarly, meaningful progress toward developing an HCV vaccine requires a better understanding of immunity that can prevent or control a hepacivirus infection. The spontaneous and timely clearance of RHV in the mouse model allows insights into the priming, expansion, and differentiation of fully-functional hepacivirus-specific T_RM_ cells. Since several studies are underway to adapt the RHV to lab mice to establish chronic infection [27,59], our results provide baseline data to understand how viruses adapt to subvert or evade immunity to develop a chronic infection in the liver. Finally, the availability of lab mice of different genetic backgrounds will enable studies to determine the importance of liver T_RM_ cells in controlling and clearing hepacivirus infection and if an effective HCV vaccine should focus on inducing robust liver T_RM_ cell immunity.

## Materials and methods

### Ethics statement

C57BL/6J mice were obtained from Jackson Laboratories (strain no. 000664) and bred under the standard protocol. Mice were 6–8 weeks of age at the time of the study. All biohazard and animal experiments were carried out in accordance with approved protocols from the Nationwide Children’s Research Institute Institutional Biosafety Committee (protocol number IBS00000285) and the Institutional Animal Care and Use Committee (protocol number AR15-00116), respectively.

### Viruses and infections

The RHV-rn1 mutant used in this study included a single amino acid substitution in NS3 protein within the CD8 T cell epitope (V970I). It was generated using site-directed mutagenesis of RHV-rn1 genome clone described earlier [23]. A mouse-adapted RHV variant was used to infect mice for reinfection and chronic infection experiments. To establish chronic RHV infection, a single dose of 500μg of anti-CD4 antibody/mouse (clone GK1.5, Bioxcell) was administered three days before RHV infection. All studies used 6–8 weeks-old mice infected intravenously via tail vein with 10^4^ to 10^5^ viral genome equivalents (VGE).

### Virus quantification

RHV titers were determined as described earlier [13] with the only modification that serum viral RNA was extracted using the Quick-RNA viral kit (ZYMO Research). In brief, viral cDNA was generated from serum-extracted RNA using the GoScript reverse transcription kit (Promega) with random hexamer priming, followed by quantification on a StepOnePlus RT-PCR system (Applied Biosystems) using the TaqMan 2x PCR master mix (Applied Biosystems). A standard curve was generated using a linearized plasmid encoding the RHV NS3 protein. The limit of detection of viral RNA was determined to be 1875 genomes/mL serum.

### Peptides

All peptides were obtained from Genemed Synthesis as a lyophilized powder. 10 mg/mL stock solutions were prepared in a 10% DMSO-water solution and stored at -80° C until use. The final concentration of each peptide in all functional assays was 2 or 10 μg/mL unless otherwise specified.

### Leukocyte isolation, culture, and cryopreservation

Isolation and culture of liver-infiltrating leukocytes were performed as described earlier [13]. Briefly, PBS-perfused livers were minced and digested with collagenase IV solution (0.01% collagenase IV in HBSS supplemented with 40 mM HEPS) for 30 min at 37°C. The cell suspension was gently homogenized through a stainless-steel mesh in HBSS supplemented with 10% FBS (Gibco). Cells were then isolated via 37% Percoll (GE Life Sciences) gradient density centrifugation at 500g for 20 min followed by lysis of residual RBCs in ACK buffer (Gibco). For cytokine stimulation assays described below, cells were cultured in RPMI-1640 containing GlutaMAX and HEPES (Gibco), 10% FBS (Gibco), 50 U/mL penicillin-streptomycin (Gibco), and 55 μM 2-mercaptoethanol (Gibco) at 37° C. For storage, cells were cryopreserved in FBS containing 10% DMSO via standard protocol.

### IFN-γ ELISpot assay

Virus-specific T cells were enumerated with the anti-mouse IFN-γ enzyme-linked immunospot (ELISPOT) assay (U-Cytech) according to the manufacturer’s protocol. Cells were cultured at 2 × 10^5^ cells per well in duplicate and stimulated with peptides, or media alone or Concanavalin-A (Sigma; 5 μg/mL) as negative and positive controls, respectively, for 40–48 h prior to plate development. The total number of spot-forming cells (SFCs) was calculated by subtracting the mean number of spots in the negative wells from the mean number of spots in test wells, followed by normalization to 10^6^ cells. A positive response was defined as >3 times the response of background wells and >50 SFCs/10^6^ cells after normalization.

### Quantification of intracellular cytokine production

For detection of RHV-specific intracellular cytokine production, one million cells were stimulated in 96-well round bottom plates with peptide(s), or media alone or PMA/Ionomycin (BioLegend) as negative and positive controls, respectively, for 5-h in the presence of GolgiPlug (BD Biosciences). Following incubation, cells were surface stained for CD3, CD4, and CD8 (20 min), fixed and permeabilized using the cytofix/cytoperm kit (BD Biosciences), and intracellularly stained for IFN-γ, TNF-α and IL-2 (30 min at RT). Dead cells were removed using the LIVE/DEAD Fixable Near-IR Dead Cell Stain kit (Invitrogen). A positive response was defined as >3 times the background staining of the negative control sample. The percentage of cytokine positive cells was then calculated by subtracting the frequency of positive events in negative control samples from that of test samples.

### Tetramer staining

Biotinylated MHC class I *H2-D*^*b*^ and class II *I-A*^*b*^ monomers specific for the RHV NS_968_ and NS3_1265_ epitopes were obtained from the NIH Tetramer Core Facility and tetramerized with streptavidin-PE (Prozyme). For direct visualization of virus-specific T cell populations, liver-infiltrating leukocytes or splenocytes were stained for 60 min at 4°C (1:500) for Class-I and 90 min at 37°C for Class-II tetramer, followed by labeling with antibodies for surface markers. Subsequently, cells were stained LIVE/DEAD Fixable Near-IR Dead Cell Stain kit, fixed, and permeabilized by transcription factor buffer set (eBiosciences) before staining for transcription factors.

### Flow cytometry data analysis and visualization

Flow cytometry data were analyzed using FlowJo software v.10.8.1. Protein expression levels were extracted as percentages and/or as MFI numerical values. Alternatively, flow cytometry data were analyzed using t-SNE visualization (Fig 3E), based on the expression levels of CD62L, CD69, PD-1, CX3CR1, and CD127.

### Cell sorting and RNA-seq library preparation

RHV-specific CD8 T cells were sorted from mice after day 100 p.i. from liver and spleen. Cells were sorted into RLT buffer and immediately frozen. RNA was extracted using an RNeasy Micro Kit (QIAGEN) according to the manufacturer’s instructions, treated with DNase I (New England Biolabs), then concentrated using Agencourt RNAClean XP beads (Beckman Coulter). Full-length cDNA and sequencing libraries were prepared using the Smart-Seq2 protocol as previously described. Libraries were loaded on two lanes of NovaSeq 6000 (Illumina) to generate replicates and 150-bp paired-end reads.

### Gene expression analysis

Sequences were analyzed using *FastQC* and mapped on the *Mus musculus* ensemble (GRCm38 release 102) reference genome using *HISAT2* (v2.2.1) [60] and transcripts were assembled using *StringTie2* (v2.1.3) [61], as described earlier [62]. Gene counts were determined using *featureCounts* of *Subread*(v2.0.0) [63]. Differential gene expression analysis was performed using *edgeR* (v3.36.0) [64] and *limma*(v3.50.3) R-packages (Bioconductor), as described earlier [65]. Genes with counts of more than two in at least two of ten samples were used to identify differentially expressed genes (DEG) between the samples and groups. The mean-variance relationship was determined using *voom* of *limma* R package. Linear modeling to fit the *log2* transformed values and weights generated by *voom* to design group was performed using *lmfit*, and *topTreat* was used to generate differential expression statistics of *limma* R package. The principal component analysis (PCA) of normalized counts was performed using *prcomp* function of *stats* R package. Pearson correlation of normalized counts was performed using *corr* function of *stats* R-package. Heatmaps were plotted using *Heatmap* function of *ComplexHeatmap* R package [66]. Volcano plot was generated using *EnhancedVolcano* R-package (Bioconductor). All RNA-seq data was submitted to NCBI Sequence Read Archive (SRA) database under BioProject ID:PRJNA99630 and SRA accession numbers SRR25336732 to SRR25336741.

### Statistical analyses

All statistical analyses were done in GraphPad PRISM 9.0.0. using a two-tailed paired or unpaired t-test, and significant p-values are shown as <0.05 (*), <0.01 (**), <0.001 (***), and <0.0001 (****).
